# Supplementary material for: ATP functions as a pathogen-associated molecular pattern to activate the E3 ubiquitin ligase RNF213
Source: Nat Commun. 2025 May 13;16:4414. doi: 10.1038/s41467-025-59444-4 (PMC12075652; doi:10.1038/s41467-025-59444-4)
Supplement: Supplementary file 1 — Supplementary Information [file 41467_2025_59444_MOESM1_ESM.pdf]

Supplementary Figure 1

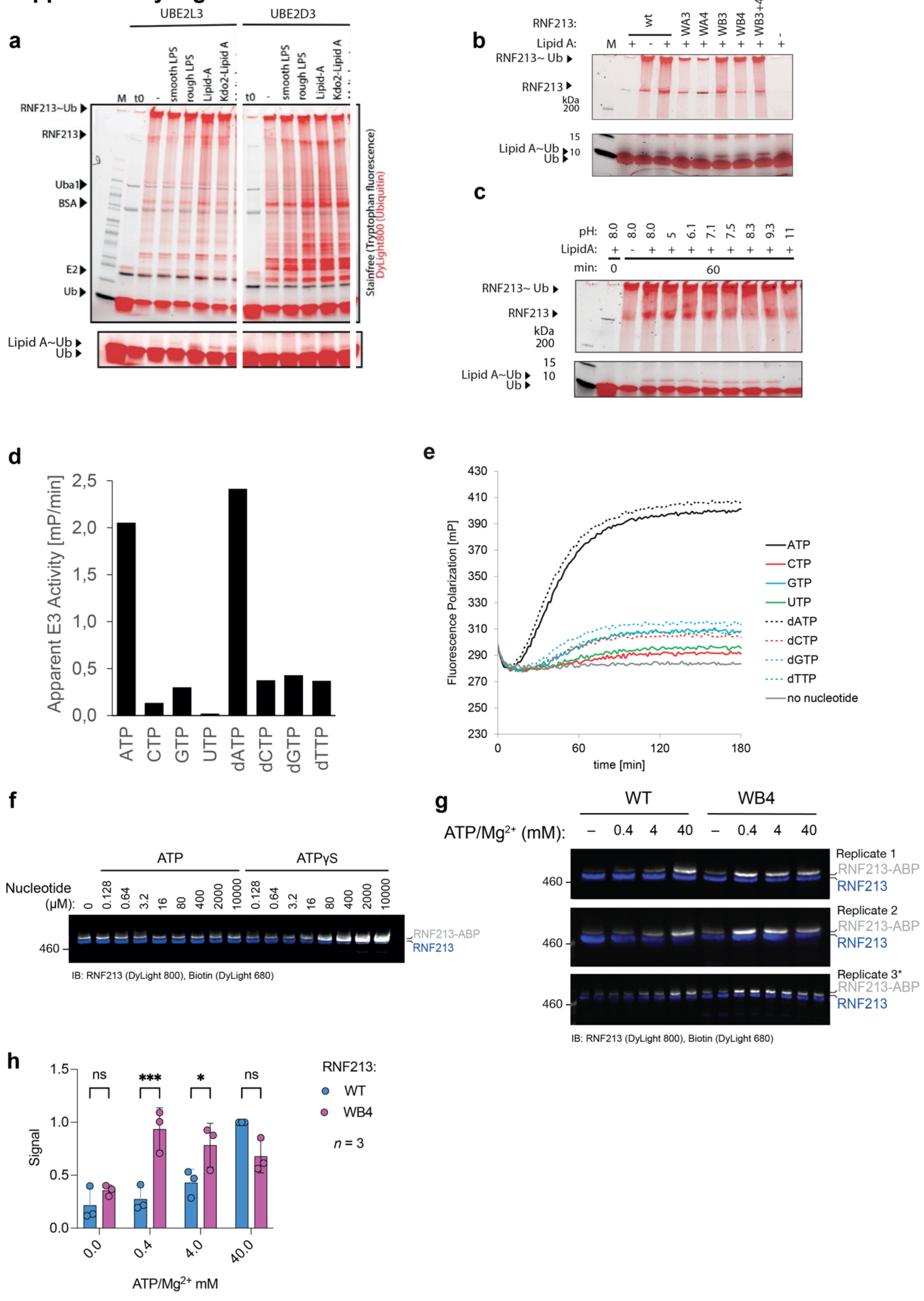

Supplementary Figure 1. **Lipid ubiquitination also requires ATP binding and RNF213 is inactive with non-adenosine nucleotides. a)** Ubiquitination assay was performed with different types of LPS as additive at a concentration of about 0.5 mg/mL. Monoubiquitination can be observed for rough LPS, Kdo2-Lipid A, and Lipid A, as reported previously. Reactivity towards LPS is preserved when UBE2L3 is replaced by UBE2D3. Smooth LPS is presumably also ubiquitinated, but the signal is not detectable due to broad distribution of smooth LPS chain lengths and the interfering signals from other ubiquitinated species in the reaction. All LPS ubiquitination assays were carried out in the presence of ATPγS, which supports E1 activity. **b)** Ubiquitination assays with RNF213 Walker A and Walker B mutants. (auto-ubiquitination, top panel; lipid A ubiquitination, lower panel). **c)** Ubiquitination assay with wild-type RNF213 in the presence of ATPγS and Lipid A. Reaction aliquots were incubated in buffers of varying pH as indicated. **d)** Reaction rates with all four canonical NTPs and dNTPs. 20 nM RNF213 was mixed with 10 μM UBE2L3~Ub and 0.2 mM of a nucleotide. Only ATP and dATP can efficiently support RNF213 activity. dATP is as efficient as ATP. Change in polarization over time is used as the measure of apparent activity. **e)** Fluorescence polarization time-course curves from which the rate was calculated. A low level of activity is observable with all nucleotides, but only ATP and dATP strongly activate RNF213. **f)** Recombinant RNF213 was tested for transthiolating activity with a biotin-tagged ABP *in vitro* in the presence of increasing concentrations of ATP or ATPγS. Samples were resolved by SDS-PAGE and visualized by two-channel near-IR immunoblot using anti-RNF213 antibody (blue) and streptavidin (white). **g)** Recombinant RNF213 and RNF213 WB4 were tested for transthiolating activity with a biotin-tagged ABP *in vitro* in the presence of increasing concentrations of ATP. * Each data point was performed in duplicate. The WB4 mutant is labelled with the probe more efficiently at low and intermediate ATP concentrations than WT. As the ABP activity signal is higher at elevated ATP concentrations, sustained activation is likely due to this displacing the inhibitory ADP product, further underscoring the importance of the effect of ATP/ADP ratio on RNF213 transthiolating activity. **h)** Quantification of ABP signals obtained in **g**, ABP signal normalized to maximal ABP labelling of WT. Two-way ANOVA, P values (left to right); 0.683 (ns), 0.0002 (***), 0.0327 (*), 0.0565 (ns); significance threshold 0.05.

Supplementary Figure 2

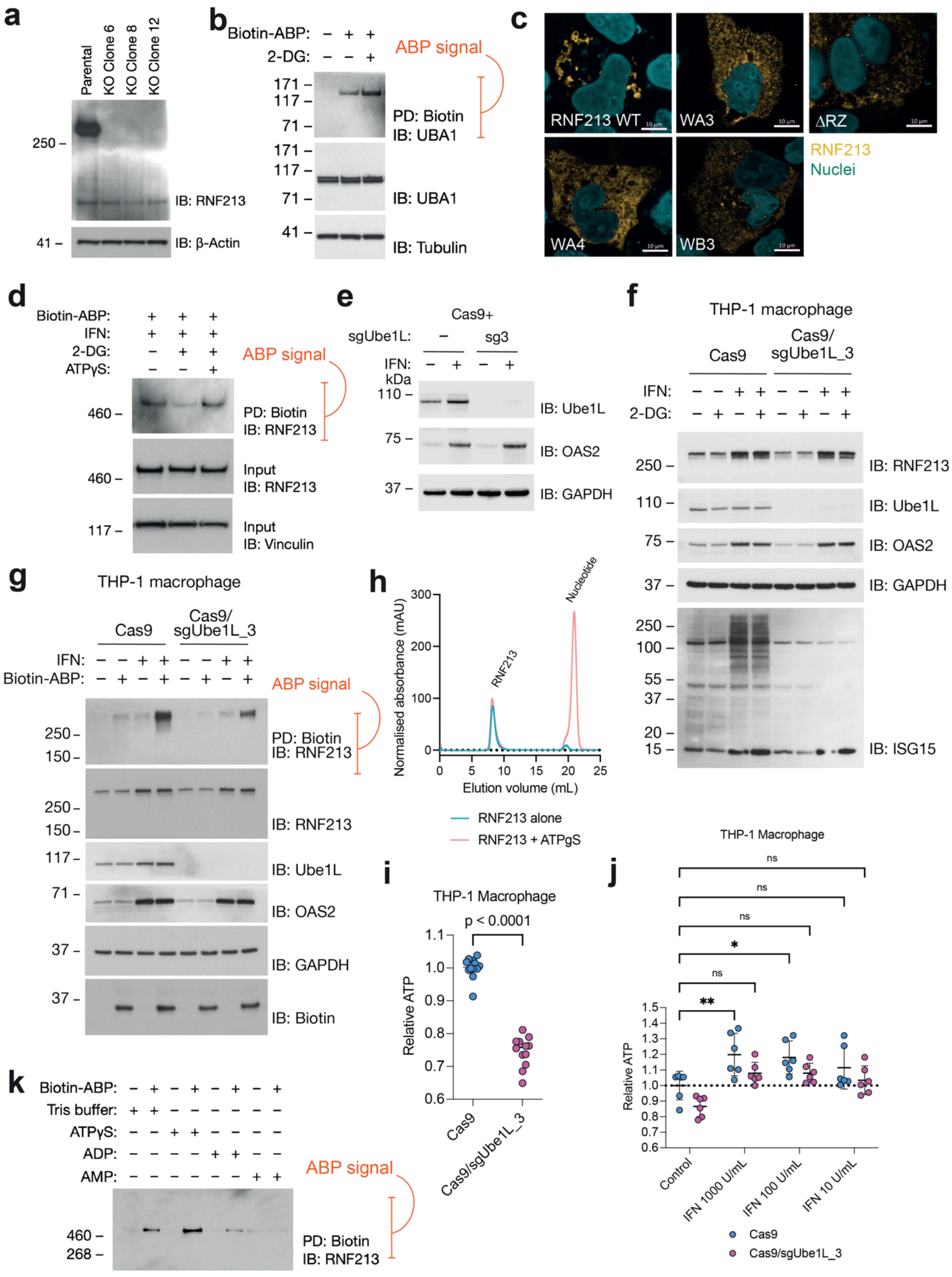

Supplementary Figure 2. Validation of RNF213 KO cell lines and *in cellula* ABP control experiments. a) Western blot detecting RNF213 or β-Actin in cell lysates from three T-REx-293 cell clones transfected with RNF213-specific sgRNA and Cas9 and selected with puromycin, and the parental T-REx-293 cell line. Predicted genetic lesions in all clones were sequence verified and clone 6 was taken forward as the RNF213-deleted cell line for reconstitution experiments. **b)** Western blot of T-REx-293$^{RNF213\ KO}$ + RNF213 WT cells pre-treated with 30 mM 2-deoxyglucose (2-DG) or vehicle control for 3 h, electroporated with biotin-ABP. Streptavidin-enriched material probed for UBA1. **c)** Immunofluorescence images of Huh-7 cells transfected with RNF213, WT or indicated variants. Huh-7 were chosen for their abundance of lipid droplets, which RNF213 is known to coat upon overexpression. All variants tested displayed altered subcellular localization when compared to the WT protein. Scale bar 10 µm. **d)** THP-1 cells treated with IFN-I overnight, with or without 2-DG for 3 h, and electroporated with biotin-ABP with or without ATPγS. Streptavidin enriched material probed with anti-RNF213 antibodies, indicative of RNF213 E3 activity. **e)** Stable depletion of Ube1L from THP-1 cells using CRIµSPR/Cas9. We screened 3 independent guide RNAs and selected one (sgUbe1L_3) which strongly depleted Ube1L. IFN stimulation of OAS2 was maintained in depleted cells. **f)** ISG15 conjugation is qualitatively unimpaired by 2-DG treatment in WT THP-1 macrophages. IFN-dependent RNF213 upregulation is unaffected in Ube1L-depleted cells. **g)** THP-1 cells treated with or without IFN-I overnight, and electroporated with a buffer control or biotin-ABP. Streptavidin enriched material probed with anti-RNF213 antibodies, indicative of RNF213 E3 activity. A reduced IFN-dependent increase in ABP signal for RNF213 is indicative of posttranslational activation of RNF213 being impaired in Ube1L-depleted cells. **h)** Size-exclusion chromatography of RNF213 alone or RNF213 pretreated with ATPγS (2 mM). Separation was carried out with a Superdex 200 10/300 GL column (Cytiva). **i)** Reduced ATP levels are found in THP-1 cells upon Ube1L depletion. Two-tailed t test, $P < 0.0001$. **j)** Quantification of ATP levels in WT and Ube1L-depleted macrophages upon IFN treatment, representative data of $n = 3$. Two-way ANOVA, P values compare all conditions to untreated control THP-1 cells (left to right); 0.0075 (**), 0.6003 (ns), 0.0175 (*), 0.5895 (ns), 0.2334 (ns), 0.9879 (ns); error bars are SD ($n = 6$). **k)** THP-1 cells electroporated with Biotin-ABP and either buffer alone, ATPγS, ADP or AMP. Streptavidin enriched material probed with anti-RNF213 antibodies.

Supplementary Figure 3

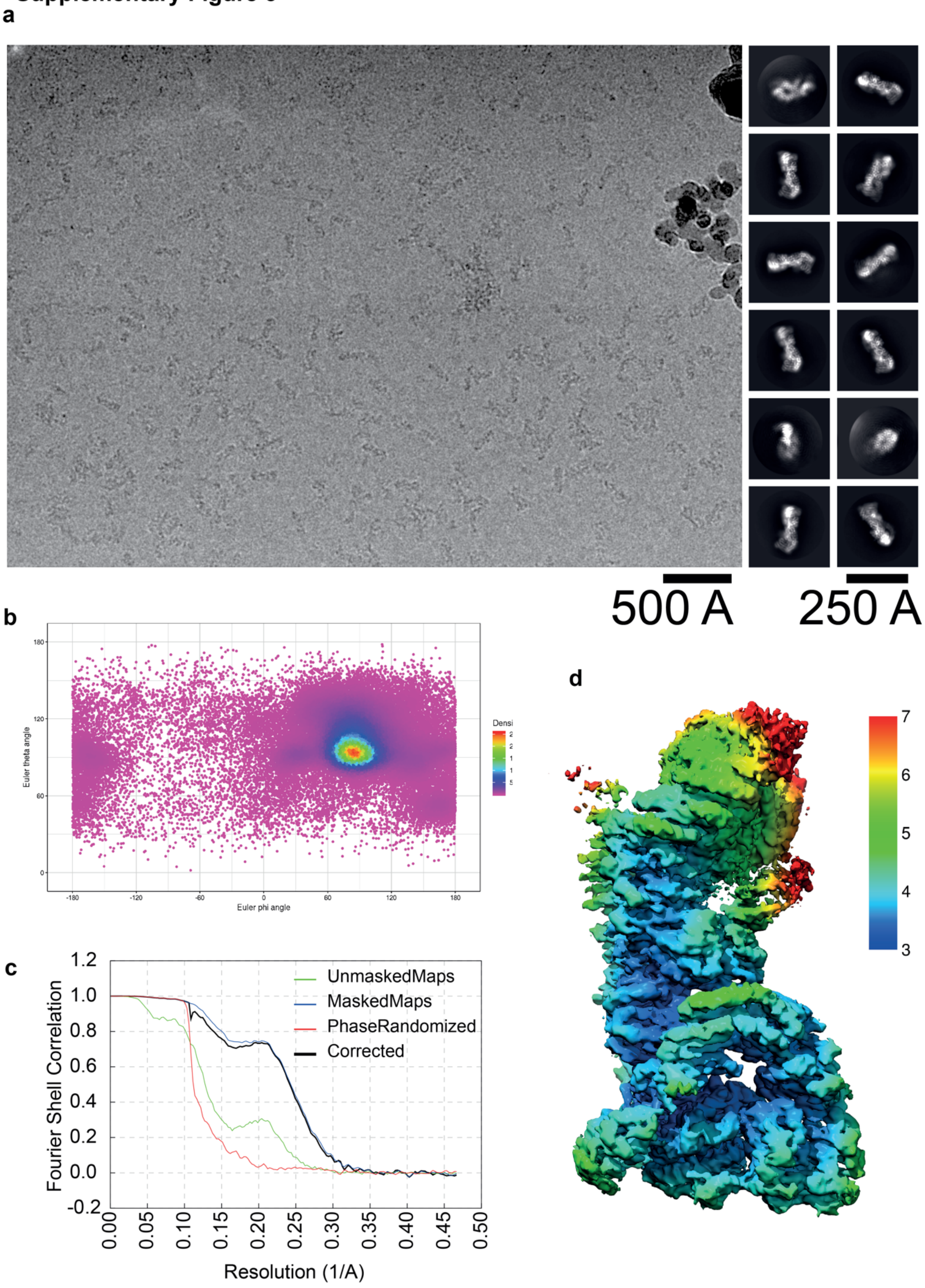

Supplementary Figure 3. **Cryo-EM analysis of the chemically stabilized RNF213:UBE2L3~Ub transthiolation intermediate. a)** Left: Example micrograph, low-pass filtered at 20 Å for visual clarity. Right: Image class averages as generated by 2D classification in relion. **b)** Angular distribution heat map of particles used to reconstruct the cryo-EM density. **c)** Fourier Shell Correlation (FSC) curves indicating a resolution of 3.5 Å using the FSC=0.143 criterion. **d)** Reconstructed cryo-EM density of the RNF213:UBE2L3~Ub complex colored by local resolution. The resolution color scale is from 3 Å (blue) to 7 Å (red).

Supplementary Figure 4

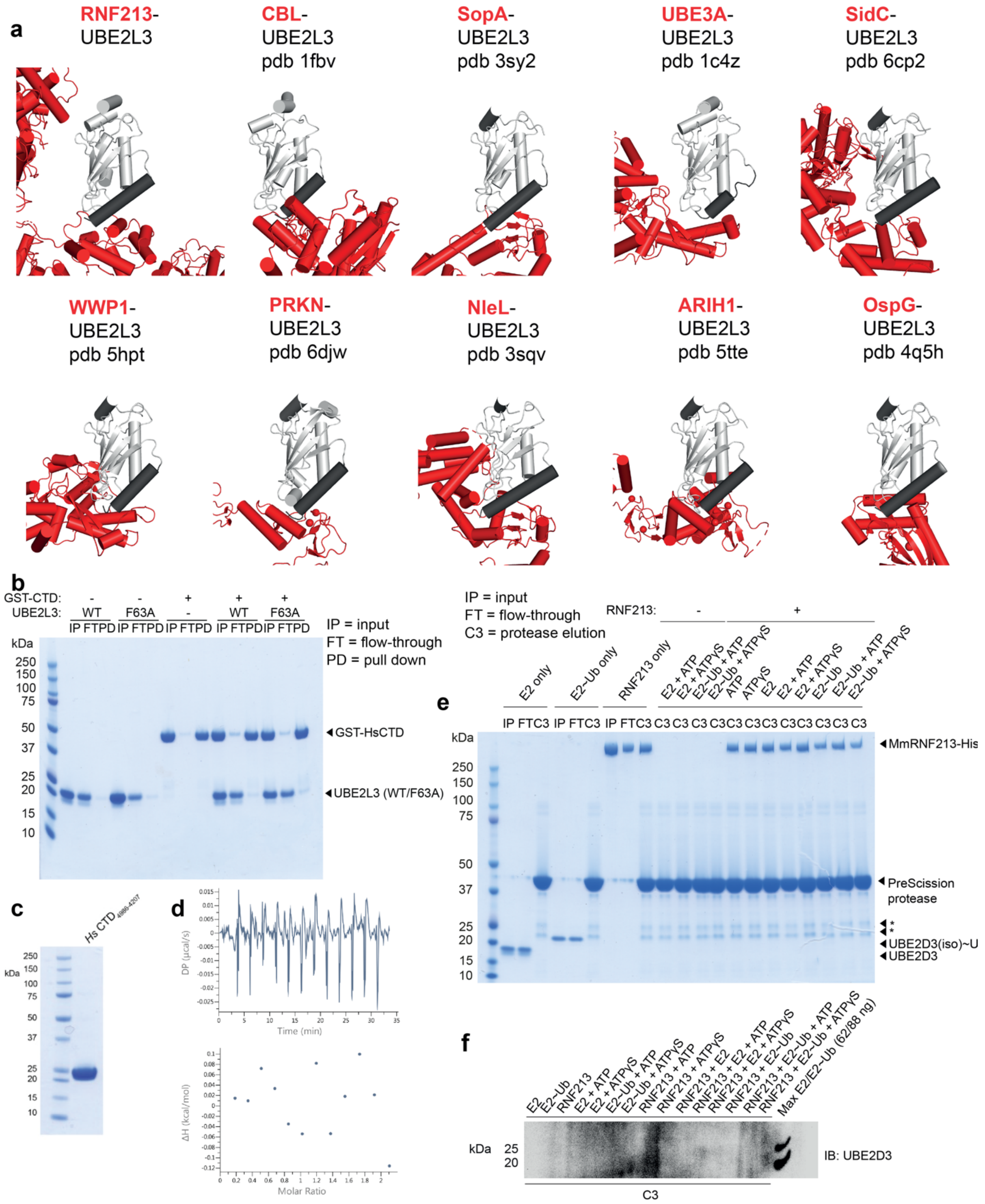

Supplementary Figure 4. Experimentally determined E2-E3 interfaces and affinity measurements between E2/E2~Ub and RNF213. a) Structural variation in published molecular models of UBE2L3-E3 complexes. Each panel shows a published structure involving an interaction between UBE2L3 (light grey) and an E3 ligase (red). Helix H1 of UBE2L3 in each structure is highlighted (dark grey). Interface between

RNF213 CTD and UBE2L3 matches the interface found in all previously known UBE2L3-E3 structures. The secondary E3-UBE2L3 interface is not found in any of the known structures, apart from the non-canonical bacterial E3s NleL and SidC, in which there exists a weak secondary interface that is similar to what appears to be the case for RNF213. **b)** Glutathione S-transferase (GST)-tagged human RNF213 CTD domain (residues 4986-5207) was expressed in *E coli.* and purified. GST-CTD pull down of UBE2L3 and a putative non-binding UBE2L3 F63A control **(Figure 4d)** was carried out. GST-CTD was bound to glutathione sepharose 4B resin and proteins were eluted with 1X LDS SDS-PAGE sample buffer. No binding was detected. Proteins were visualized by Coomassie staining. **c)** For biophysical measurements the GST tag was cleaved from GST-CTD and the protein purified. **d)** Isothermal titration calorimetry (ITC) was used to measure binding between the human CTD domain and UBE2L3. UBE2L3 was placed in the cell (70 µM) and CTD was placed in the syringe (715 µM). No binding was detected, indicating it lacks a measurable enthalpy change or the dissociation constant (K_d) is > ~500 µM (based on simulated isotherms using the experimental concentrations). **e)** Pull down of the E2 UBE2D3 and a stabilized mimic of its E2~Ub conjugate by full length mouse StrepII-tagged RNF213 was carried out. Proteins were combined and RNF213 was immobilized on streptactin resin. To elute RNF213, the resin was incubated with precision protease. No pull down was detected, even in the presence of nucleotides (5 mM). Asterisks correspond to contaminants in the PreScission protease sample. **f)** Pull down of UBE2D3 or $UBE2D3_{isopeptide}$~Ub following rhinovirus 3C protease treatment was also assessed by immunoblotting against UBE2D3. No pulldown was detected. The last lane is a reference consisting of a mixture of UBE2D3 and $UBE2D3_{isopeptide}$~Ub representing stoichiometric binding to the RNF213 input (62 and 88 ng, respectively).

Supplementary Figure 5

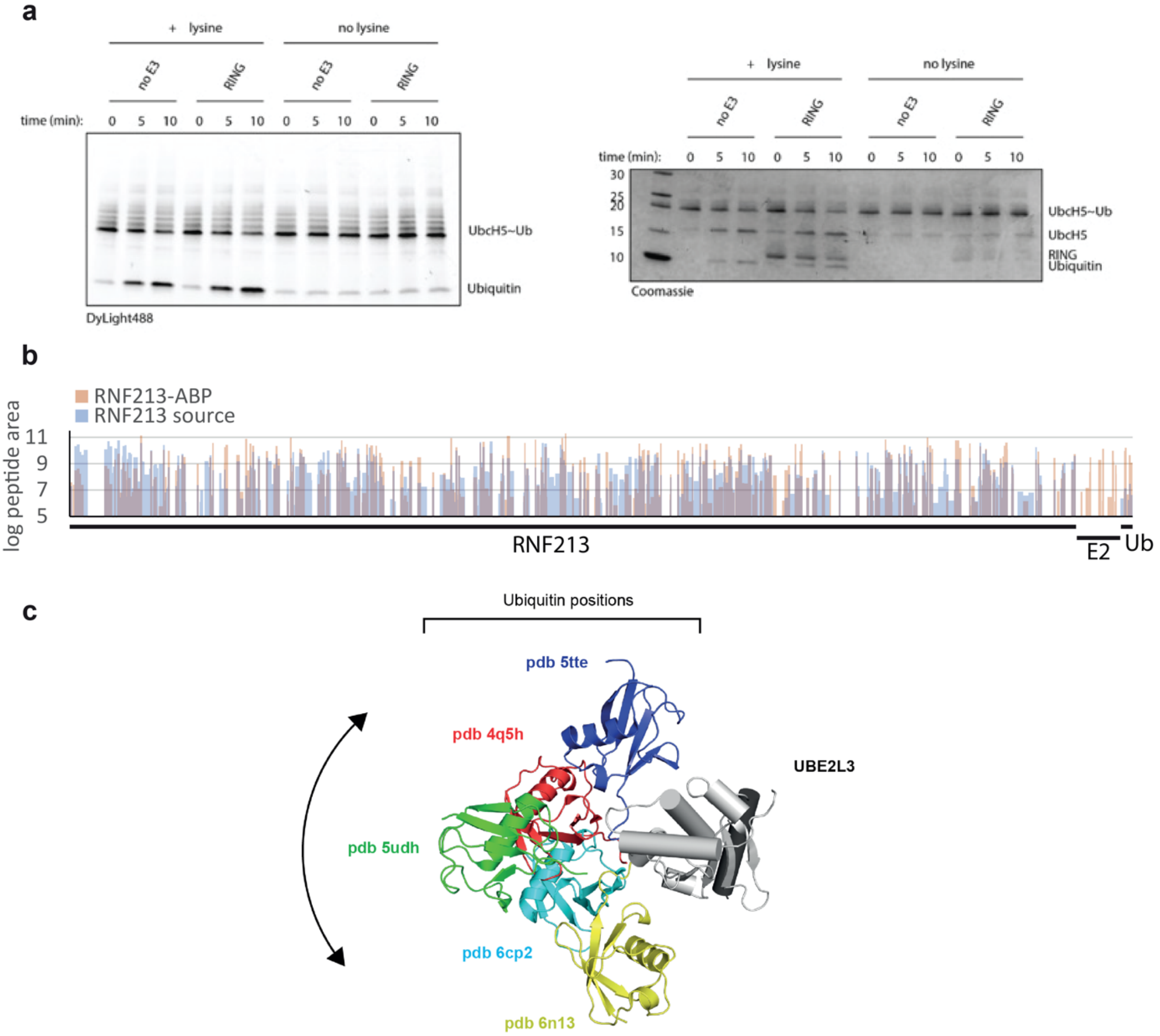

Supplementary Figure 5. RNF213 RING E3 activity, components of the RNF213-ABP complex and dynamics of the E2~Ub conjugate. a) The ability of the RNF213 RING domain to stimulate discharge of Ub from an E2~Ub to free lysine (20 mM), a characteristic of archetypical RING domains, was tested. Reaction was performed under single turnover conditions (UbcH5~Ub (5 µM) and His-SUMO-RNF213$_{RING}$ (5 µM)**.** Left, signal from Dylight488 (Ub); right, Coomassie stain. **b)** Peptide coverage for different components of the RNF213-ABP complex, in the source RNF213 material and after coupling to ABP, following purification by SEC. Value at each residue is the sum of signals (label-free quantification) of all PSMs covering that residue. Log$_{10}$ of the value shown in the plot. Trace amounts of Ub peptides detectable in the source

material. E2 and Ub peptide coverage in the RNF213-ABP sample suggests a 1:1:1 adduct. **c)** Position of ubiquitin (in color) relative to UBE2L3 (light grey) in known structures. Ub does not have a unique defined position relative to UBE2L3, indicating it is likely that the Ub moiety on RNF213 is also flexible. Helix H1 of UBE2L3 is highlighted in dark grey.

Supplementary Figure 6

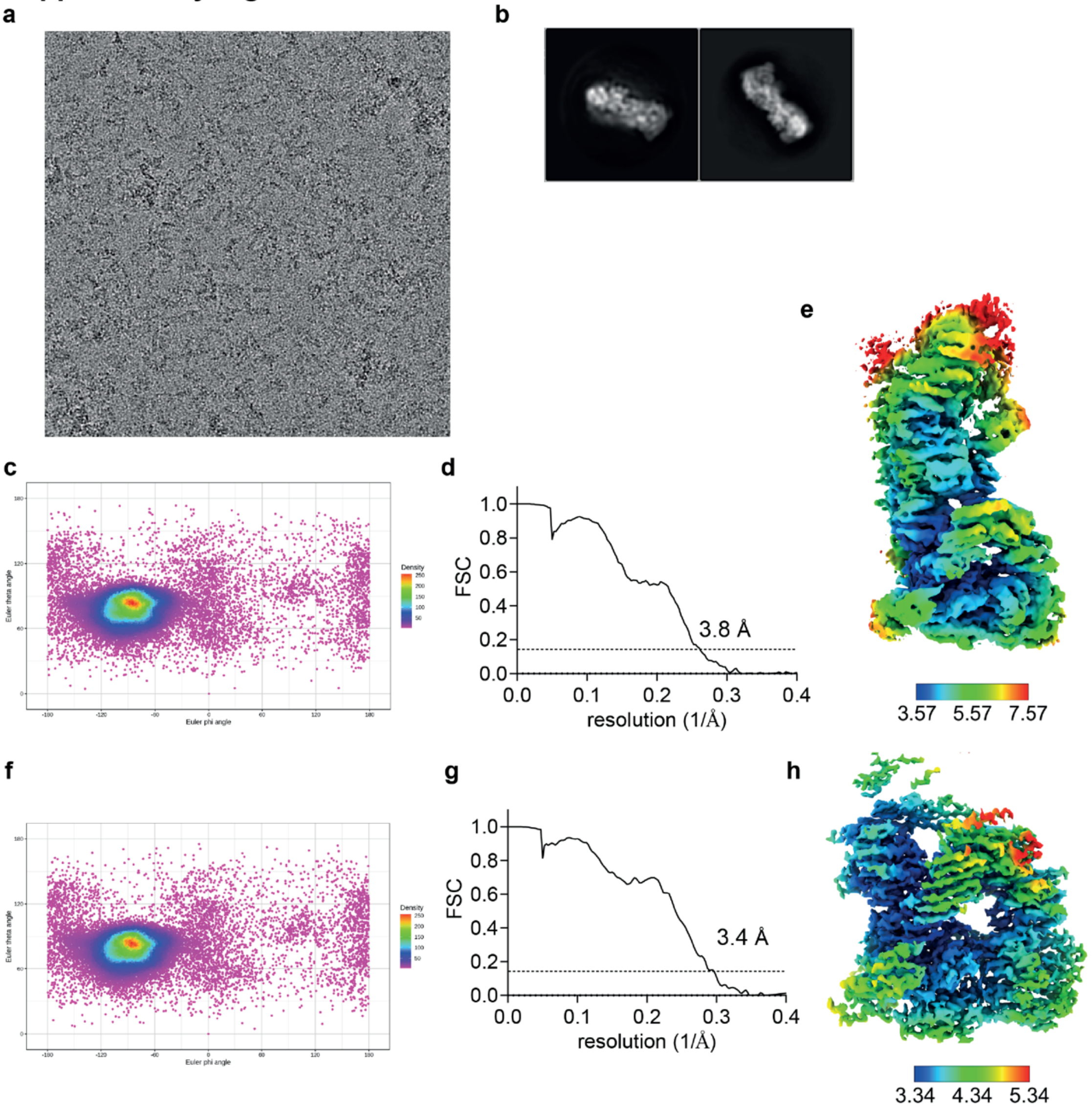

Supplementary Figure 6. **Cryo-EM analysis of RNF213-ABP in complex with ADP. a)** Example micrograph, low-pass filtered for visual clarity. **b)** Image class averages as generated by 2D classification in Relion. **c)** Angular distribution heat map of particles used to reconstruct the cryo-EM density. **d)** Fourier Shell Correlation (FSC) curves indicating a resolution of 3.8 Å using the FSC=0.143 criterion. **e)** Reconstructed cryo-EM density colored by local resolution. The resolution color scale is indicated. **f)** Angular distribution heat map of particles used to calculate a focused cryo-EM density map of the AAA core. **g)** Fourier Shell Correlation (FSC) curves indicating a resolution of 3.4 Å using the FSC=0.143 criterion. **h)** Reconstructed cryo-EM density colored by local resolution. The resolution color scale is indicated.

Supplementary Figure 7

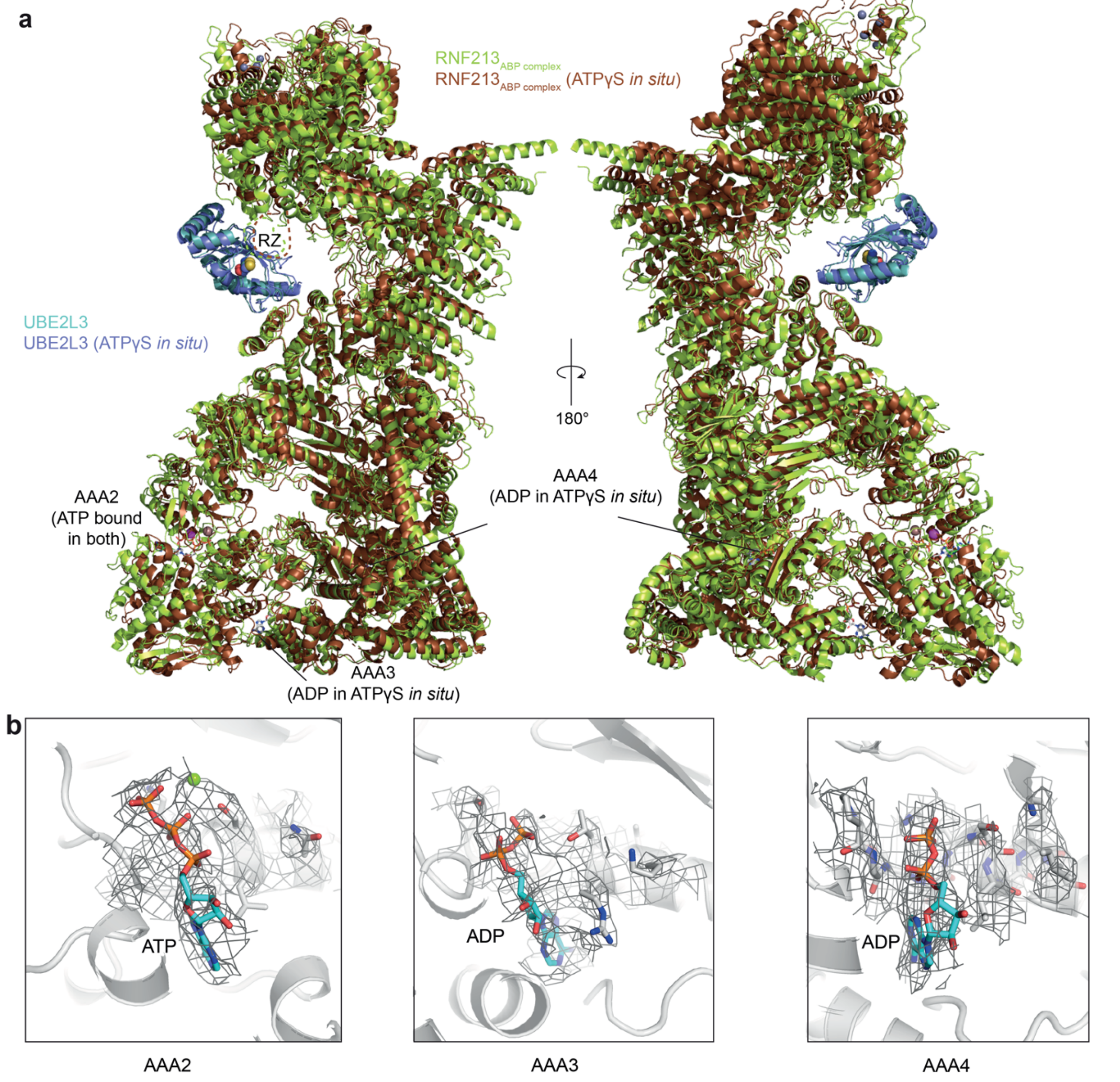

Supplementary Figure 7. Structural alignment of CryoEM RNF213-ABP complex with second RNF213-ABP CryoEM model obtained with ATPγS *in situ*. a) The structure obtained from applying the unpurified ABP reaction (ATPγS *in situ*) to cryo-EM grids underwent modest structural rearrangement (RMSD = 2.66 Å) relative to the original structure. Alignments were carried out with PyMOL 3.0.0 (Schrödinger). **b)** As consistently observed before, density corresponding to ATP was present at the AAA2 site that lacks Walker B residues required for hydrolysis. The *in situ* ATPγS conditions produced density consistent with ADP being bound to sites AAA3 and AAA4.

Supplementary Figure 8

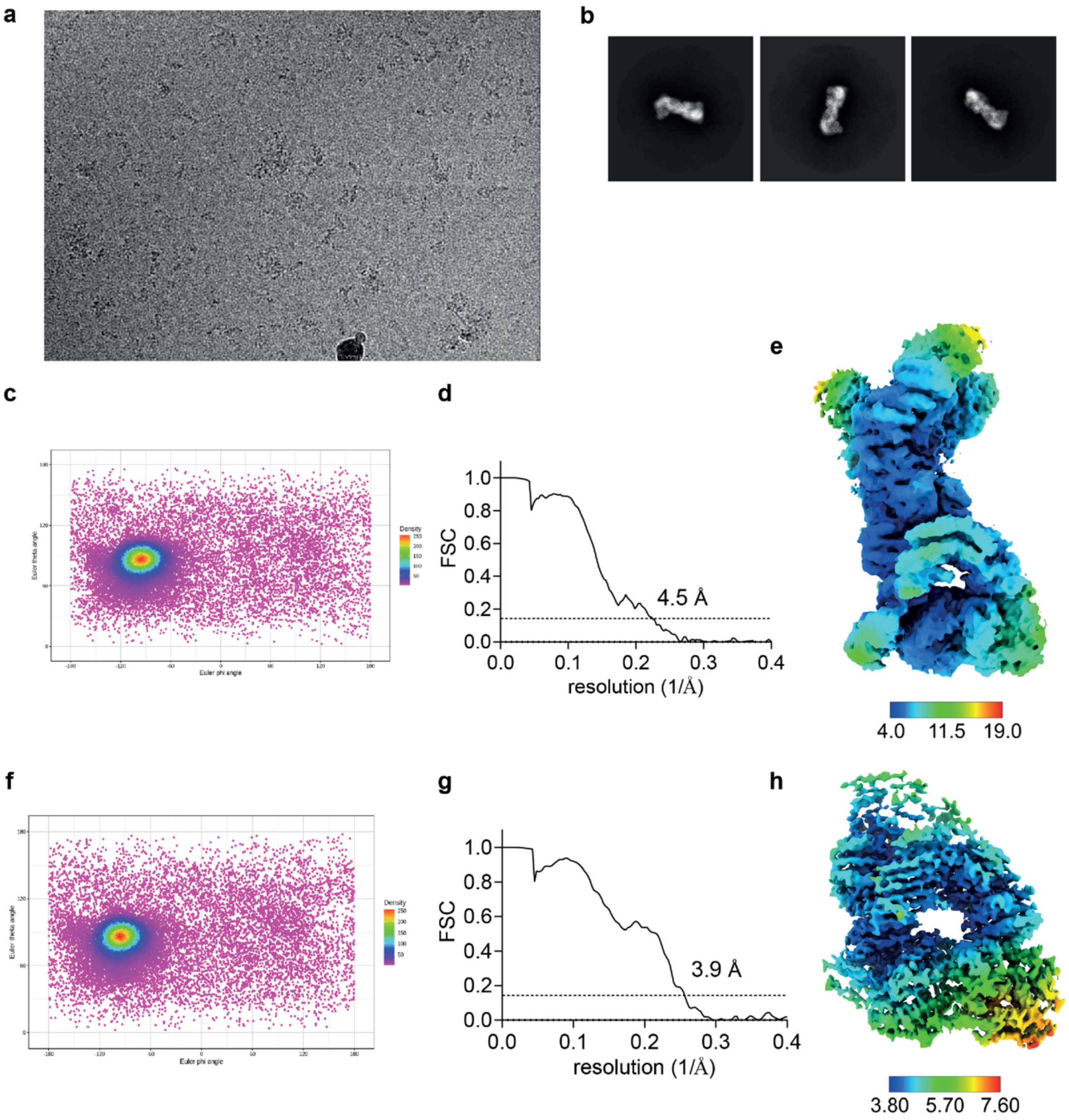

Supplementary Figure 8. Cryo-EM analysis of the WB34 mutant of RNF213 in complex with ATP. a) Example micrograph, low-pass filtered for visual clarity. **b)** Image class averages as generated by 2D classification in Relion. **c)** Angular distribution heat map of particles used to reconstruct the cryo-EM density. **d)** Fourier Shell Correlation (FSC) curves indicating a resolution of 4.5 Å using the FSC=0.143 criterion. **e)** Reconstructed cryo-EM density colored by local resolution. The resolution color scale is indicated. **f)** Angular distribution heat map of particles used to calculate a focused cryo-EM density map of the AAA core. **g)** Fourier Shell Correlation (FSC) curves indicating a resolution of 3.9 Å using the FSC=0.143 criterion. **h)**

Reconstructed cryo-EM density colored by local resolution. The resolution color scale is indicated.

Supplementary Figure 9

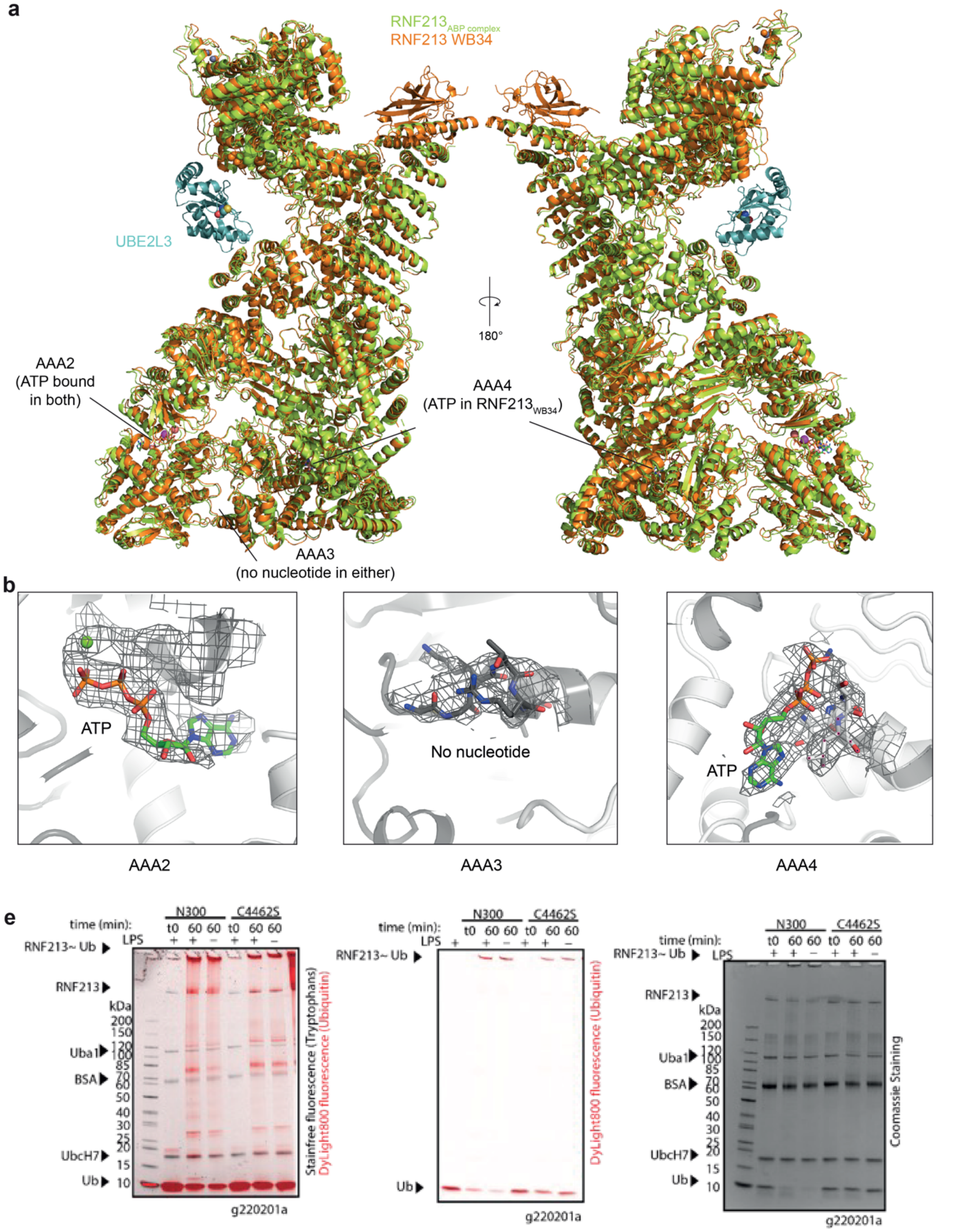

Supplementary Figure 9. Structural alignment of CryoEM RNF213-ABP complex with RNF213 WB34 CryoEM model in the presence of ATP and attempts to trap an RNF213 catalytic intermediate. a) The hydrolysis defective RNF213 WB34 mutant remained similar to the structure of RNF213 in the original RNF213-ABP complex (RMSD = 1.57 Å). Alignments were carried out with PyMOL 3.0.0

(Schrödinger). **b)** As consistently observed before, density corresponding to ATP was present at the AAA2 site that lacks Walker B residues required for hydrolysis. The WB34 mutation allowed trapping of ATP in the AAA4 site but the AAA3 site remained empty. **c)** Autoubiquitination reactions for RNF213 ΔN300 and RNF213 C4462S. Although LPS ubiquitination is prevented with the C4462S mutation, it retains autoubiquitination activity. This background signal masks the potential formation of a stabilized ester-linked linked intermediate. Left, stainfree fluorescence (protein bands), and overlaid dylight fluorescence (labelled ubiquitin); center, dylight fluorescence; right Coomassie stain.

Supplementary Figure 10

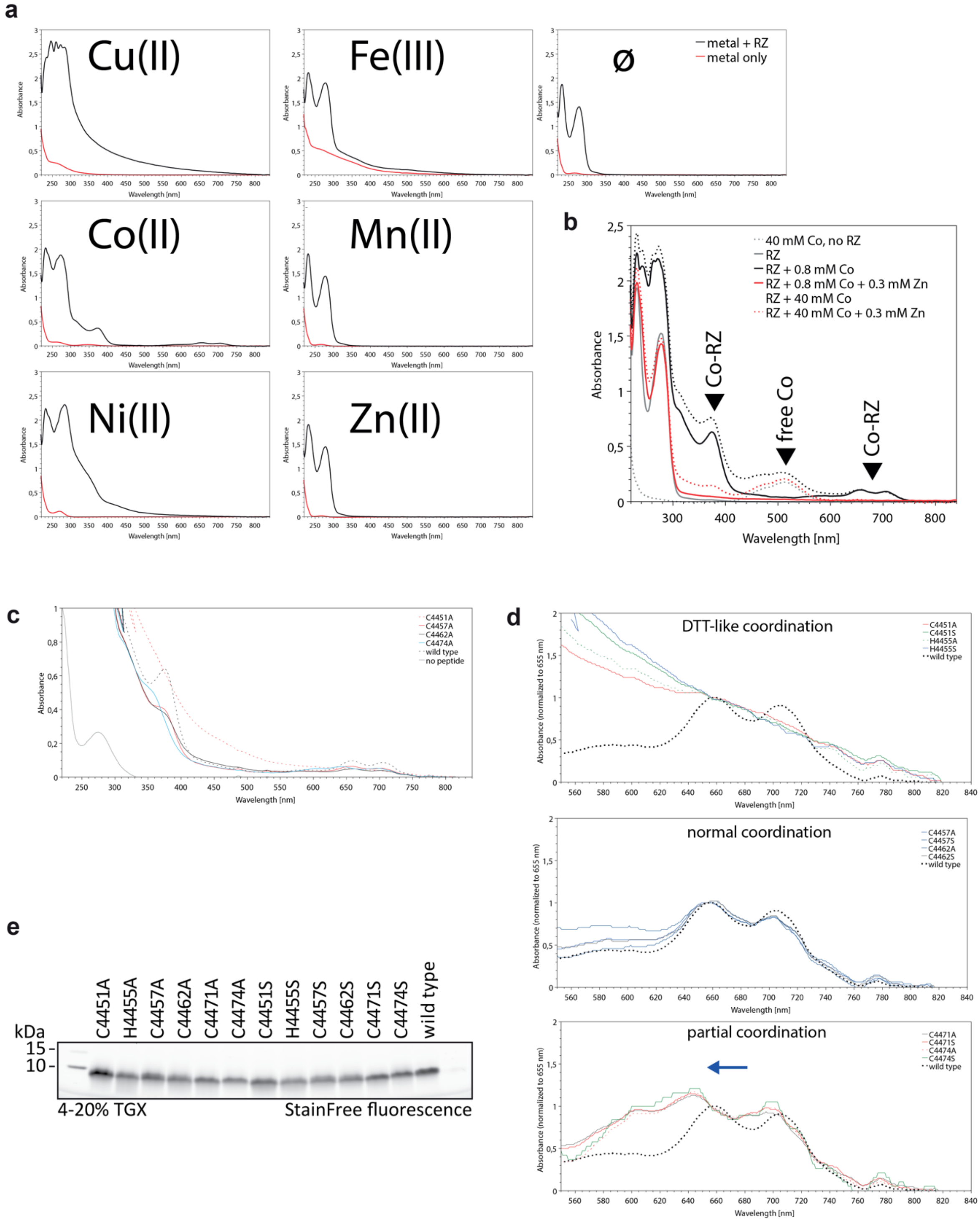

Supplementary Figure 10. **Metal binding in the RZ domain. a)** A synthetic peptide corresponding to the core RNF213 RZ domain is able to bind divalent metal ions of Cu(II), Co(II), and Ni(II), but does not bind the trivalent Fe(III). Binding of Mn(II) and Zn(II) cannot be directly assessed as they do not absorb in the measured UV-VIS

wavelength range. **b)** Zinc(II) can out-compete bound Co(II) even when a >100-fold excess of Co(II) over Zn(II) is present. The Co(II)-specific spectral signal is greatly diminished, indicating a strong preference of the RNF213 RZ domain for zinc over Co(II). Peaks of interest are indicated with a black triangle. **c-d)** Alanine and serine point substitutions of all residues possibly involved in metal binding in the RNF213 RZ motif were synthetized and their solutions were prepared in the RNF213 buffer at 200 µM with a 1.5-fold excess of $CoCl_2$. (c) Full UV-VIS spectra of the formed cobalt complexes of RNF213 RZ alanine mutants of all sites. Altered spectra can be observed for C4451A, H4455A, C4471A, and C4474A, while C4457A and C4462A appear similar to the wild type. (d) Detailed view of the spectra of the RZ-Co complexes, zoomed in on the characteristic Co-zinc-finger complex peak between 600 nm and 800 nm. Three groups of mutations can be observed. Mutations of Cys4451 and His4455 lead to a complete loss of the characteristic peak and to a Co-DTT-like spectrum, giving the solutions a slightly brown tinge. Mutations of Cys4457 and Cys4462 match the spectrum of the wild type. Finally, mutations of Cys4471 or Cys4474 retain the specific Co-zinc-finger peak, but with a clear blueshift, consistent with incomplete coordination. The alanine and serine mutants of each residue match perfectly, making it highly unlikely that the observed effects are non-specific. **e)** SDS-PAGE analysis of the peptides used in the metal binding assay.

Supplementary Figure 11

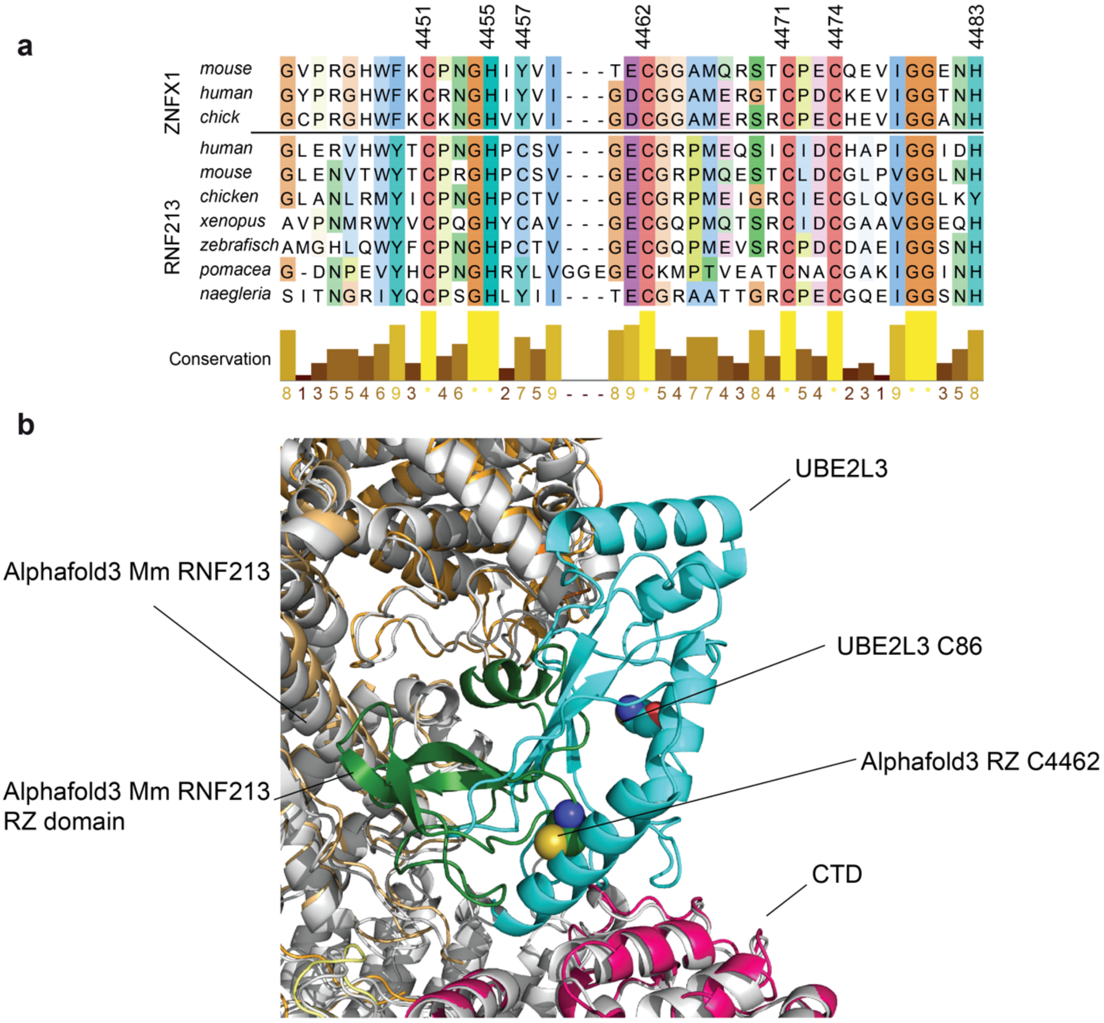

Supplementary Figure 11. Multiple sequence alignment of RZ domain proteins, and alignment of RZ domain containing AlphaFold3 RNF213 model with our RNF213-ABP complex. a) The residues determined to be involved in Zn^{2+} binding are fully conserved, as is the active site Cys4462. Jalview conservation score is displayed below the alignment. **b)** An AlphaFold3 model of mouse RNF213 (lacking the N-terminal 340 residues) contained a modeled RZ domain. However, alignment of the model to the CTD domain in our ABP stabilized RNF213-E2 complex results in substantial clash between the RZ domain and the E2. This suggests the RZ domain can adopt multiple positions and must reorient for cysteine juxtaposition, required for transthiolation.
