# Supplementary material for: ATP functions as a pathogen-associated molecular pattern to activate the E3 ubiquitin ligase RNF213
Source: Nat Commun. 2025 May 13;16:4414. doi: 10.1038/s41467-025-59444-4 (PMC12075652; doi:10.1038/s41467-025-59444-4)
Supplement: Supplementary file 2 — Reporting Summary [file 41467_2025_59444_MOESM2_ESM.pdf]

nature portfolio

Corresponding author(s): Adam Fletcher
Satpal Virdee
Tim Clausen

Last updated by author(s): Mar 31, 2025

Reporting Summary

Nature Portfolio wishes to improve the reproducibility of the work that we publish. This form provides structure for consistency and transparency in reporting. For further information on Nature Portfolio policies, see our Editorial Policies and the Editorial Policy Checklist.

Statistics

For all statistical analyses, confirm that the following items are present in the figure legend, table legend, main text, or Methods section.

n/a	Confirmed	
☐	☒	The exact sample size (n) for each experimental group/condition, given as a discrete number and unit of measurement
☐	☒	A statement on whether measurements were taken from distinct samples or whether the same sample was measured repeatedly
☐	☒	The statistical test(s) used AND whether they are one- or two-sided<br>*Only common tests should be described solely by name; describe more complex techniques in the Methods section.*
☐	☒	A description of all covariates tested
☐	☒	A description of any assumptions or corrections, such as tests of normality and adjustment for multiple comparisons
☐	☒	A full description of the statistical parameters including central tendency (e.g. means) or other basic estimates (e.g. regression coefficient) AND variation (e.g. standard deviation) or associated estimates of uncertainty (e.g. confidence intervals)
☐	☒	For null hypothesis testing, the test statistic (e.g. F, t, r) with confidence intervals, effect sizes, degrees of freedom and P value noted<br>*Give P values as exact values whenever suitable.*
☒	☐	For Bayesian analysis, information on the choice of priors and Markov chain Monte Carlo settings
☒	☐	For hierarchical and complex designs, identification of the appropriate level for tests and full reporting of outcomes
☒	☐	Estimates of effect sizes (e.g. Cohen's d, Pearson's r), indicating how they were calculated

Our web collection on statistics for biologists contains articles on many of the points above.

Software and code

Policy information about availability of computer code

Data collection	The RNF213 and ABP protein samples were recorded on a Titan Krios G3 instrument equipped with a Gatan BioQuantum Energy Filter and a Gatan K3 camera.
Data analysis	Cryo-EM data processing was carried out in Relion 3.1, with particle picking carried out by a combination of Cryolo and Relion 3D template picking using the model generated by Cryolo-picked particles. Images of the cryo-EM density were generated by ChimeraX. Densitometry of Western blots was performed with Fiji Image J. Quantification of fluorescent Western blots was performed using Licor software Empira. Confocal immunofluorescence images were processed with the Zen software.

For manuscripts utilizing custom algorithms or software that are central to the research but not yet described in published literature, software must be made available to editors and reviewers. We strongly encourage code deposition in a community repository (e.g. GitHub). See the Nature Portfolio guidelines for submitting code & software for further information.

Data

Policy information about availability of data

All manuscripts must include a data availability statement. This statement should provide the following information, where applicable:

- Accession codes, unique identifiers, or web links for publicly available datasets
- A description of any restrictions on data availability
- For clinical datasets or third party data, please ensure that the statement adheres to our policy

Atomic coordinates and cryo-EM density map have been deposited in the Protein Data Bank (PDBe) and EMDB databases under accession codes 7OIK, EMD-12931

for the structure of the RNF213-ABP adduct. The raw micrographs were submitted to the EMPIAR database (EMPIAR-10711). The mass spectrometry proteomics data have been deposited to the ProteomeXchange Consortium via the PRIDE86 partner repository with the dataset identifier PXD025992 (for reviewers, Username: reviewer_pxd025992@ebi.ac.uk; Password: 3XZBazmA). All other source data are included with the paper.

Research involving human participants, their data, or biological material

Policy information about studies with human participants or human data. See also policy information about sex, gender (identity/presentation), and sexual orientation and race, ethnicity and racism.

Reporting on sex and gender	*Use the terms sex (biological attribute) and gender (shaped by social and cultural circumstances) carefully in order to avoid confusing both terms. Indicate if findings apply to only one sex or gender; describe whether sex and gender were considered in study design; whether sex and/or gender was determined based on self-reporting or assigned and methods used. Provide in the source data disaggregated sex and gender data, where this information has been collected, and if consent has been obtained for sharing of individual-level data; provide overall numbers in this Reporting Summary. Please state if this information has not been collected. Report sex- and gender-based analyses where performed, justify reasons for lack of sex- and gender-based analysis.*
Reporting on race, ethnicity, or other socially relevant groupings	*Please specify the socially constructed or socially relevant categorization variable(s) used in your manuscript and explain why they were used. Please note that such variables should not be used as proxies for other socially constructed/relevant variables (for example, race or ethnicity should not be used as a proxy for socioeconomic status). Provide clear definitions of the relevant terms used, how they were provided (by the participants/respondents, the researchers, or third parties), and the method(s) used to classify people into the different categories (e.g. self-report, census or administrative data, social media data, etc.) Please provide details about how you controlled for confounding variables in your analyses.*
Population characteristics	*Describe the covariate-relevant population characteristics of the human research participants (e.g. age, genotypic information, past and current diagnosis and treatment categories). If you filled out the behavioural & social sciences study design questions and have nothing to add here, write "See above."*
Recruitment	*Describe how participants were recruited. Outline any potential self-selection bias or other biases that may be present and how these are likely to impact results.*
Ethics oversight	*Identify the organization(s) that approved the study protocol.*

Note that full information on the approval of the study protocol must also be provided in the manuscript.

Field-specific reporting

Please select the one below that is the best fit for your research. If you are not sure, read the appropriate sections before making your selection.

☒ Life sciences ☐ Behavioural & social sciences ☐ Ecological, evolutionary & environmental sciences

For a reference copy of the document with all sections, see nature.com/documents/nr-reporting-summary-flat.pdf

Life sciences study design

All studies must disclose on these points even when the disclosure is negative.

Sample size	For the mass spectometry experiments 6 replicates of each conditions were included.
Data exclusions	No data points were excluded.
Replication	In general triplicate experiments were performed on cell and in vitro biochemical assays unless otherwise indicated.
Randomization	Cell genotypes were assigned as indicated.
Blinding	Cell culture expreiments were not blinded; samples were analysed with the cell genotype or experimental condition known. Mass spectrometry experiments were performed on coded samples so the operator was not aware of sample identity.

Reporting for specific materials, systems and methods

We require information from authors about some types of materials, experimental systems and methods used in many studies. Here, indicate whether each material, system or method listed is relevant to your study. If you are not sure if a list item applies to your research, read the appropriate section before selecting a response.

Materials & experimental systems

n/a	Involved in the study
[ ]	[x] Antibodies
[ ]	[x] Eukaryotic cell lines
[x]	[ ] Palaeontology and archaeology
[x]	[ ] Animals and other organisms
[x]	[ ] Clinical data
[x]	[ ] Dual use research of concern
[x]	[ ] Plants

Methods

n/a	Involved in the study
[x]	[ ] ChIP-seq
[x]	[ ] Flow cytometry
[x]	[ ] MRI-based neuroimaging

Antibodies

Antibodies used

The primary antibodies used were: anti-RNF213 (HPA026790, Rabbit, Merck); anti-β-Actin (Cell Signaling Technology, #3700); anti-Biotin-HRP (Cell Signaling Technology #7075); anti-UBA1 (Thermo Fisher PA5-81869); anti-RNF213 (obtained by inoculating rabbits with peptide INELKVFVDLASISAGEND coupled to KLH carrier and purifying total immunoglobulin from blood serum, Eurogentec); anti-Ube1L (polyclonal, 15818-1-AP, Proteintech); anti-ISG15 (Polyclonal, Cell Signaling Technology, 2743); anti-OAS2 (Polyclonal, 19279-1-AP, Proteintech); anti-GAPDH (Invitrogen, AM4300); Streptavidin Protein, DyLight™ 680 (Thermo Fisher 21848)

Validation

All commercial antibodies were used according to manufacturers instructions and not validated, except anti-RNF213, validated in CRISPR KO cells (Sup Fig 2)

Eukaryotic cell lines

Policy information about cell lines and Sex and Gender in Research

Cell line source(s)

T-REx-293 Flp-In cells were a gift from Alfredo Castello and originally purchased from Thermo Fisher Scientific. THP-1 cells and Huh-7 cells were a gift from Sam Wilson and John McLauchlan respectively.

Authentication

None of the cell lines used were authenticated.

Mycoplasma contamination

Cell lines were routinely tested and found negative for mycoplasma

Commonly misidentified lines
(See ICLAC register)

Name any commonly misidentified cell lines used in the study and provide a rationale for their use.

Plants

Seed stocks

Report on the source of all seed stocks or other plant material used. If applicable, state the seed stock centre and catalogue number. If plant specimens were collected from the field, describe the collection location, date and sampling procedures.

Novel plant genotypes

Describe the methods by which all novel plant genotypes were produced. This includes those generated by transgenic approaches, gene editing, chemical/radiation-based mutagenesis and hybridization. For transgenic lines, describe the transformation method, the number of independent lines analyzed and the generation upon which experiments were performed. For gene-edited lines, describe the editor used, the endogenous sequence targeted for editing, the targeting guide RNA sequence (if applicable) and how the editor was applied.

Authentication

Describe any authentication procedures for each seed stock used or novel genotype generated. Describe any experiments used to assess the effect of a mutation and, where applicable, how potential secondary effects (e.g. second site T-DNA insertions, mosiacism, off-target gene editing) were examined.
